# Aortic Stenosis Complicated by Gastrointestinal Arteriovenous Malformations: It is not Always Heyde Syndrome

**DOI:** 10.7759/cureus.8876

**Published:** 2020-06-28

**Authors:** Ravi A Thakker, Krishna H Suthar, Kevin Kline, Khaled Chatila, Muhannad Al Hanayneh

**Affiliations:** 1 Internal Medicine, University of Texas Medical Branch, Galveston, USA; 2 Gastroenterology and Hepatology, University of Texas Medical Branch, Galveston, USA; 3 Cardiology, University of Texas Medical Branch, Galveston, USA

**Keywords:** aortic stenosis, arteriovenous malformations, acquired von willebrand syndrome, heyde syndrome

## Abstract

Aortic stenosis (AS) and arteriovenous malformations (AVM) are a common coexisting pathology in the elderly. When both pathologies are combined, Heyde syndrome is a differential that is widely explored among clinicians. Unfortunately, this may not always be the case. We present a case of an 82-year-old female admitted for acute gastrointestinal (GI) bleeding with a history of AVMs and AS, as well as, an algorithm in diagnosing elderly patients with both pathologies.

## Introduction

Heyde syndrome is a complex syndrome involving multiple organ systems. It is characterized by aortic stenosis (AS), gastrointestinal (GI) bleeding, and acquired von Willebrand syndrome (aVWS). The exact prevalence of this syndrome is unknown due to the independent prevalence of AS and GI bleeding. Some studies have shown that in patients with moderate to severe AS about 1%-3% had notable GI bleeding, as well as, a reduction in von Willebrand factor (vWF) in approximately 20%-70% of patients with AS [1]. In the elderly, a combination of both arteriovenous malformations (AVM) and moderate to severe AS has been an important hallmark as a clinical presentation of Heyde syndrome. On the contrary, this may not always be the case. We present a case of an 82-year-old female admitted for acute GI bleeding with a history of AVM and AS. We also outline an algorithm in diagnosing patients with GI bleeding and AS.

## Case presentation

**Figure 1:**
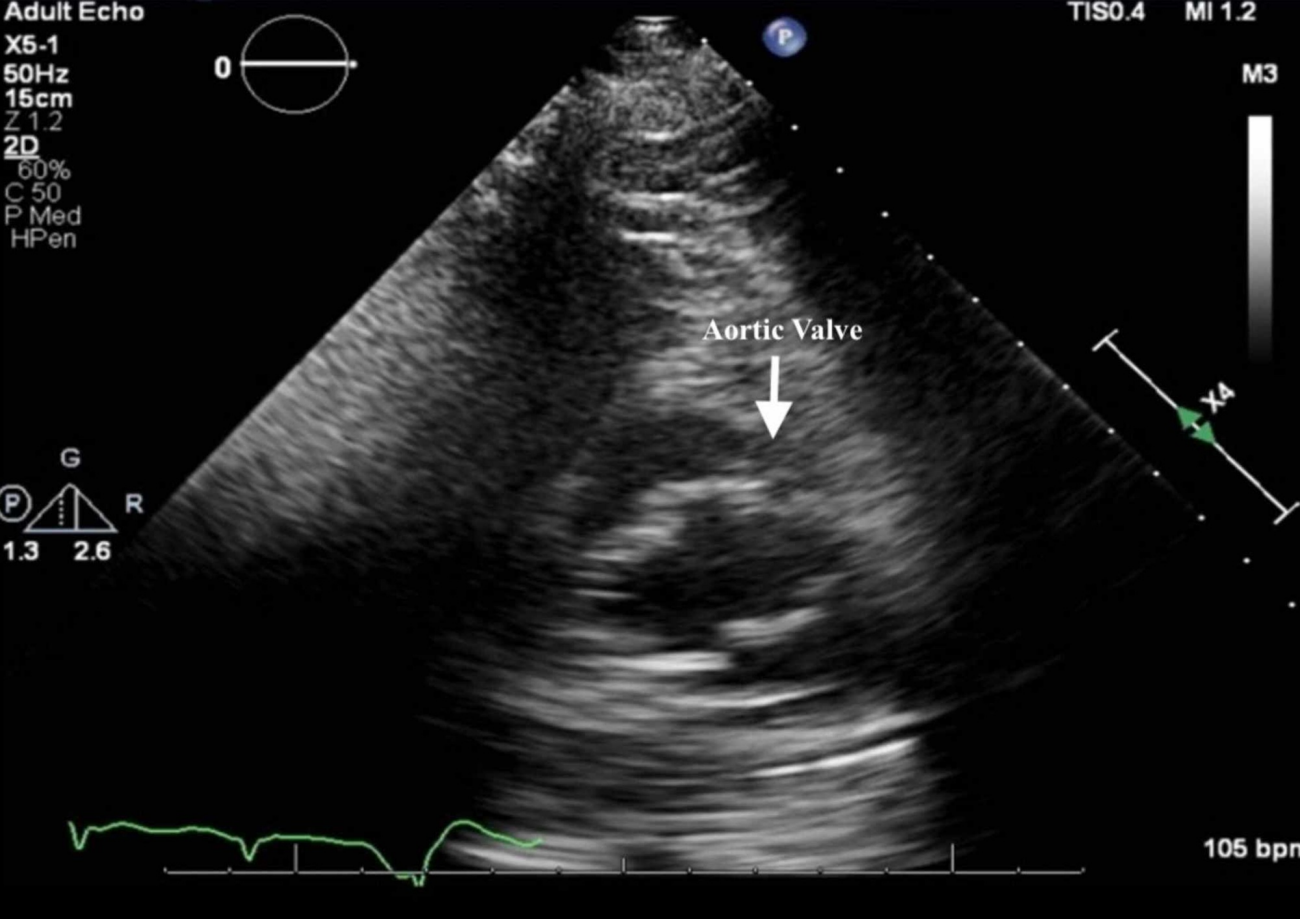
TTE short axis view demonstrating aortic stenosis. TTE, transthoracic echocardiogram

Our patient is an 82-year-old Caucasian female with past medical history of heart failure with reduced ejection fraction of 40%-45%, low flow low gradient moderate to severe AS, persistent hypotension with chronic oral vasopressor use, coronary artery disease with percutaneous coronary intervention, paroxysmal atrial fibrillation not on anticoagulation due to bleeding from prior cecal and ascending colon AVMs with subsequent argon plasma coagulation (APC) treatment, chronic kidney disease, and chronic obstructive pulmonary disease who presented to the hospital for fatigue in the context of a hemoglobin level of 6.2 g/dL. At that time, she was also noted to have evidence of heart failure exacerbation with bibasilar crackles and pedal edema noted on physical examination. During her hospital course she developed atrial fibrillation with rapid ventricular response and was transferred to the cardiac critical care unit with eventual rate control through digoxin and amiodarone. During her course, she was discussed in a multi-disciplinary manner between heart failure and interventional cardiology services as well as cardiothoracic surgery for potential balloon aortic valvuloplasty and subsequent transcatheter aortic valve replacement (TAVR). Transthoracic echocardiogram (TTE) demonstrated low flow low gradient moderate to severe AS with an aortic valve area between 0.64 and 1 cm^2^ and mean aortic valve gradient measures between 22 and 33.9 mmHg (Figure 1).

Left heart catheterization demonstrated areas between 1.22 and 1.42 cm^2^. The procedure was postponed as there was low concern for severe AS after repeat TTE and left heart catheterization. She was transferred back to the intermediate cardiac care unit with an optimized rate control for atrial fibrillation, heparin drip for anticoagulation bridging, and diuresis for her heart failure exacerbation. Of note, she also had elevated vWF activity on laboratory workup. During her first night on the floor she suffered an episode of melena with drop in her hemoglobin. Her activated partial thromboplastin time (aPTT) was noted at that time to be supratherapeutic, and the heparin drip was discontinued. She responded appropriately to transfusion. She remained on proton pump inhibitor (PPI) and octreotide drip with eventual discontinuation of both and transition to oral PPI. She had a gradual rise in her hemoglobin appreciated throughout her stay with a resolution of active GI bleeding.

## Discussion

**Figure 2:**
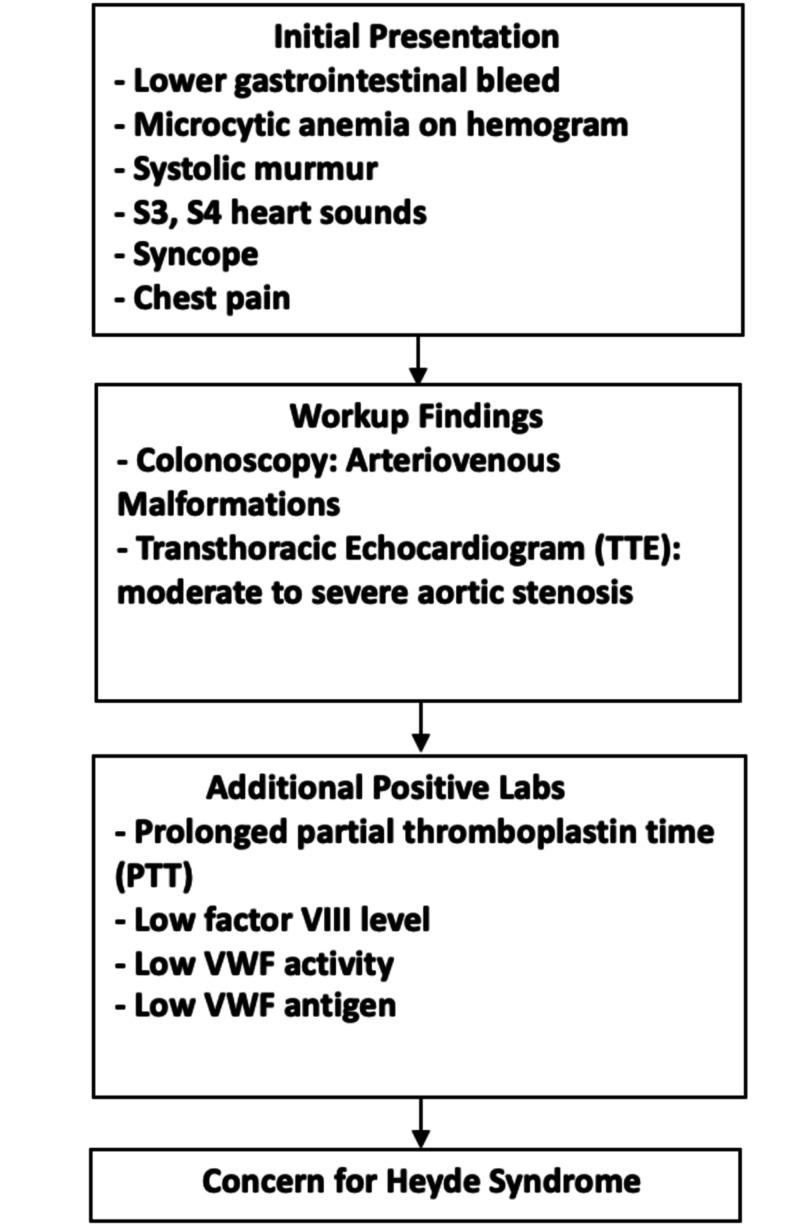
Algorithm for aortic stenosis and GI bleed. GI, gastrointestinal

There are many theories on the pathophysiology of Heyde syndrome. The most commonly recognized theory revolves around platelet trauma and poor clotting. Increased shear across the stenosed aortic valve results in unfolding of vWF and activation by the metalloprotease ADAMTS 13. In turn, there is an exacerbated interplay between vWF and platelets with resultant premature removal of vWF [2]. Approximately 21% of patients with aVWS will have shear-induced activation of ADAMTS13 such as AS, hypertrophic cardiomyopathy, ventricular septal defect, left ventricular assist device, or pulmonary hypertension [3]. With regard to laboratory findings, an average of 20%-70% of moderate to severe AS patients will have increased removal of high molecular weight vWF [4]. There are a wide variety of GI pathologies that affect the elderly. With regard to upper GI bleeding, peptic ulcer disease has been shown to be one of the most common pathologies. Pathologies known to be common mainly in the elderly include aortoenteric fistulas and gastric antral vascular ectasia. Colonic diverticula has been shown to be among the most common pathology of lower GI bleeding in the elderly population. Other notable lower GI bleeding pathologies include angiodyplasia and benign neoplasm [5]. The exact prevalence of AS in the elderly is unknown but has shown to range up to 22.8% among North American and Europeans aged 75 and older [6]. Our algorithm outlines an approach to diagnosing patients presenting with AS and GI bleeding (Figure 2).

Our case report highlighted a unique case of a patient with low flow low gradient moderate to severe AS with an underlying history of AVM and elevated vWF activity. Initial concern was for Heyde syndrome in context of her classic presentation. She did not undergo aortic valve replacement with conservative management done through close hemodynamic monitoring, resuscitation, and PPI drip with gradual clinical improvement.

## Conclusions

Key points from our case are the importance of considering for AS in elderly patients with history of AVM, as well as, keeping a broad approach in evaluating patients presenting with a history of AS and acute GI bleeding.
